# Chronic Iron Overload Results in Impaired Bacterial Killing of THP-1 Derived Macrophage through the Inhibition of Lysosomal Acidification

**DOI:** 10.1371/journal.pone.0156713

**Published:** 2016-05-31

**Authors:** Jun-Kai Kao, Shih-Chung Wang, Li-Wei Ho, Shi-Wei Huang, Shu-Hao Chang, Rei-Cheng Yang, Yu-Yuan Ke, Chun-Ying Wu, Jiu-Yao Wang, Jeng-Jer Shieh

**Affiliations:** 1 Institute of Biomedical Sciences, National Chung Hsing University, Taichung, Taiwan; 2 Pediatric Department, Children’s Hospital, Changhua Christian Hospital, Changhua, Taiwan; 3 Department of Education and Research, Taichung Veterans General Hospital, Taichung, Taiwan; 4 Institute of Clinical Medicine, National Yang Ming University, Taipei, Taiwan; 5 Department of Pediatrics, Taichung Veterans General Hospital, Taichung, Taiwan; 6 Division of Gastroenterology and Hepatology, Taichung Veterans General Hospital, Taichung, Taiwan; 7 Allergy and Clinical Immunology Research (ACIR) Center, College of Medicine, National Cheng Kung University, Tainan, Taiwan; 8 China Medical University Children’s Hospital, Taichung, Taiwan; 9 Rong Hsing Research Center for Translational Medicine, National Chung Hsing University, Taichung, Taiwan; Charles University in Prague, CZECH REPUBLIC

## Abstract

Iron is essential for living organisms and the disturbance of iron homeostasis is associated with altered immune function. Additionally, bacterial infections can cause major complications in instances of chronic iron overload, such as patients with transfusion-dependent thalassemia. Monocytes and macrophages play important roles in maintaining systemic iron homoeostasis and in defense against invading pathogens. However, the effect of iron overload on the function of monocytes and macrophages is unclear. We elucidated the effects of chronic iron overload on human monocytic cell line (THP-1) and THP-1 derived macrophages (TDM) by continuously exposing them to high levels of iron (100 μM) to create I-THP-1 and I-TDM, respectively. Our results show that iron overload did not affect morphology or granularity of I-THP-1, but increased the granularity of I-TDM. Bactericidal assays for non-pathogenic *E*. *coli* DH5α, JM109 and pathogenic *P*. *aeruginosa* all revealed decreased efficiency with increasing iron concentration in I-TDM. The impaired *P*. *aeruginosa* killing ability of human primary monocyte derived macrophages (hMDM) was also found when cells are cultured in iron contained medium. Further studies on the bactericidal activity of I-TDM revealed lysosomal dysfunction associated with the inhibition of lysosomal acidification resulting in increasing lysosomal pH, the impairment of post-translational processing of cathepsins (especially cathepsin D), and decreased autophagic flux. These findings may explain the impaired innate immunity of thalassemic patients with chronic iron overload, suggesting the manipulation of lysosomal function as a novel therapeutic approach.

## Introduction

Iron, an essential nutrient for most living organisms, is involved in several cellular functions, such as oxygen transportation and energy production. Iron homeostasis requires a complex regulation system that is not yet well understood [1]. Iron homeostasis disturbances, especially iron overload, are associated with chronic diseases such as atherosclerosis, metabolic syndrome, hepatitis, Alzheimer’s disease, and cancer [2]. Iron also influences the immune system; iron supplementation has been reported to increase susceptibility to malaria and tuberculosis [3–5]. Bacterial infections cause major complications in cases of chronic iron overload, such as in patients with transfusion-dependent thalassemia. Studies focusing on the effects of chronic iron overload on the immune system have demonstrated that iron overload is associated with defective chemotaxis and phagocytosis of neutrophils and macrophages as well as decreased bactericidal activity, contributing to decreased immune function [6–10]. However, the results of these studies are inconsistent and the mechanisms are still unclear.

Vertebrate host defense against microbes represents the integration of innate and acquired immune systems, which together respond to a diverse array of infectious threats [11, 12]. Monocytes/macrophages (part of the reticuloendothelial system) are major elements of the innate immune system. Following stimulation by inflammation, monocytes migrate to tissues and differentiate into macrophages that function in both, nonspecific defense and specific antigen presentation. Specific pattern recognition receptors “recognize” specific pathogens, followed by an activated signal transduction cascade that triggers proinflammatory responses and phagocytosis [13]. Internalization of the phagocytic particle is followed by phagosome maturation and eventual fusion with the lysosome, a cytoplasmic membrane-enclosed organelle containing hydrolytic enzymes that degrade macromolecules and cell components, to form a phagolysosome [14]. Following phagocytosis, pathogens are subjected to a variety of killing mechanisms within activated macrophages. Before fusing with the lysosome, phagocytosed material is immediately exposed to cytotoxic reactive oxygen species (ROS). After a transient increase in phagolysosomal pH, the phagolysosome is acidified to a pH ≦ 5.0 to activate digestive lysosomal enzymes that efficiently kill phagocytosed organisms. Several degradation pathways converge at the lysosomal level, including endocytosis, phagocytosis, and autophagy. The first two pathways degrade components from the extracellular milieu, while autophagy mainly degrades intracellular components [15]. Both heterophagy and autophagy are associated with pathogen defense [16]. In addition to defense against invading pathogens, macrophages are critical for mammalian iron homeostasis. The phagocytosis of senescent erythrocytes and their degradation by macrophages enables efficient recycling of iron and maintenance of iron homeostasis [2]. This dual role of monocyte/macrophages leads to an assumption that they are most susceptible to iron among all immune cells.

Since limited information is available regarding the effects of iron on immunity [17], we aimed to elucidate the effect of chronic iron exposure on cell models of monocyte/macrophage systems, THP-1 cells (human monocytic cell line) and THP-1 derived macrophages (TDM), in this study. The results of this study provide insights into impaired innate immunity in thalassemic patients with chronic iron overload.

## Materials and Methods

### Reagents and antibodies

Ferrous sulfate heptahydrate (FeSO_4_), desferrioxamine (DFO), phorbol 12-myristate 13-acetate (PMA), chloroquine (CQ) and bafilomycin A1 (BafA1) were obtained from Sigma (St. Louis, MO). Antibodies specific for LC3 and p62 were purchased from Novus (Littleton, CO). Antibodies specific for LAMP1, LAMP2, cathepsin B (CTSB) and cathepsin D (CTSD) were purchased from Cell Signaling Technology (Danvers, MA). The antibody specific for CD11c and CD86 was purchased from eBioscience (San Diego, CA). The antibody specific for TFEB and β-actin was purchased from Santa Cruz Biotechnology (Santa Cruz, CA).

### Cell culture

Human monocytic leukemia THP-1 (ATCC: TIB-202, Rockville, MA) and mouse macrophage cell line RAW264.7 (ATCC: TIB-71) were maintained in RPMI 1640 and DMEM media, respectively, supplemented with 10% fetal bovine serum (FBS), penicillin G (100 IU/ml), streptomycin (100 μg/ml), and L-glutamine (2 mM) and incubated at 37°C. TDM cells were obtained by treating THP-1 monocytes with 25 ng/ml PMA for 48 h in 6-well culture plates (Greiner, Germany) with 2 ml cell suspension (5 × 10^5^ cells) in each well. Differentiated cells were washed and incubated for another 24 h in the culture medium to return to the resting state of macrophages (M0). For chronic iron overload experiments, THP-1, TDM and RAW264.7 cells were persistently maintained and subcultured in media containing 100 μM FeSO_4_.

### Isolation of human monocytes and differentiate them into macrophages

This study was approved by the Ethics Committee of Changhua Christian Hospital, Taiwan (CCH IRB No. 120102). All subjects gave written consent to participate in this study. Human PBMC was separated first from anti-coagulated human blood by density gradient centrifugation using Ficoll-Paque^™^ PLUS (GE Healthcare, Uppsala, Sweden). Human monocytes were then purified from Prepared PBMC using BD IMag^™^ Anti-Human CD14 Magnetic Particles—DM (BD Biosciences, San Diego, CA), which positive selects CD14^+^ monocytes. Isolated human monocytes were cultured in RPMI 1640, supplemented with 10% heat-inactivated FCS (CSL Biosciences, Parkville, VIC, Australia), 2 mM GlutaMax-1 (Invitrogen, Carlsbad, CA), 100 U/ml penicillin, and 100 mg/ml streptomycin, and stimulated with human GM-CSF (5 ng/ml) for 7 d to differentiate them into macrophages [18].

### Cell viability assays

The percent of viable cells was calculated as the number of cells that were not stained by trypan blue divided by the total number of cells within the grids of the hemocytometer. Alternatively, cells were harvested and aliquoted into tubes in densities of up to 1 × 10^6^ cells/100 μl. Propidium iodide (PI) was added (0.5–1 μl) to each sample immediately prior to analysis. PI fluorescence was measured using the FL-2 or FL-3 channel with a Cytomics^™^ FC500 Flow Cytometer (Beckman Coulter, Fullerton, CA). Cell viability under the test conditions was reported as a percentage relative to untreated cells.

### Flow cytometry analysis

TDM and I-TDM cells were suspended in phosphate-buffered saline (PBS) containing 2% FBS. After blocking non-specific antibody binding with Fc block antibody, fluorochrome-labeled antibodies specific for macrophage surface markers were added. Cells were stained on ice and washed three times with cold PBS. Unstained samples were prepared for cell size and granularity assessments. Data were collected and analyzed using Cytomics^™^ FC500 flow cytometer.

### Reactive oxygen species assays

The generation of ROS was evaluated by intracellular oxidation of 2′,7′-dichlorofluorescein diacetate (DCFDA) staining (Molecular Probes, Eugene, OR). Cells were incubated overnight in normal culture media. The medium was replaced with fresh medium containing different concentrations of iron for 12 h. After ion exposure, the cells were washed with PBS, resuspended at a concentration of 1 × 10^6^ cells/ml, and stained by DCFDA for 30 min at 37°C. Stained cells were detected and analyzed using cytomics^™^ FC500 flow cytometer.

### Phagocytosis assay

Macrophage phagocytic activity was measured using Vybrant phagocytosis assay kit (Invitrogen, Carlsbad, CA). Briefly, THP-1 and I-THP-1 cells were differentiated into TDM and I-TDM cells via incubation with PMA for 48 h and subsequent starvation for 10 h. The cells in four replicates were stimulated with agonists for 2 h with or without pre-treatment with inhibitors. The cells were further incubated with heat-inactivated, fluorescein-labeled *Escherichia coli* K-12 BioParticles for 2 h. Extracellular fluorescence was quenched by trypan blue and phagocytic activity was quantified by measuring the fluorescence intensity of the phagocytosed particles at an emission wavelength of 520 nm and an excitation wavelength of 480 nm using an EnSpire Multilabel Reader 2300 (PerkinElmer, Waltham, MA).

### Bactericidal assay

Bactericidal activity was measured using the gentamicin protection assay with balanced salt solution (BSS) buffer. Briefly, 2.5 × 10^6^ cells or cells treated with 50 μM CQ were plated in dishes and exposed to nonpathogenic *E*. *coli* DH5α, JM109 (2.5 × 10^6^ CFU/dish, Invitrogen) or opportunistic pathogen *Pseudomonas aeruginosa* (ATCC 27853, 2.5 × 10^6^ CFU/dish). After incubation for 20 min at 37°C, cells were washed with cold BSS and then treated with gentamicin to eliminate extracellular bacteria. After washing all traces of gentamicin with cold BSS, the cells were incubated at 37°C for 0 h, 2 h, 4 h and 8 h, after which they were lysed in an equal volume of lysis buffer. The entire cell lysates from each time point was plated on MacConkey agar plates and incubated overnight. Bactericidal activity was quantified in term of colony-forming units at each time point.

### Iron content analysis

The effect of iron uptake on THP-1 and TDM cells was analyzed using Iron Assay Kit (Abcam, Cambridge, MA), as per the manufacturer instruction. Cells were harvested and washed twice with PBS and centrifugated at 300×*g*. The cell pellets were lysed by adding a 10-fold volume of iron assay buffer, centrifuged at 16000×*g* for 10 min, and the supernatant was collected. Iron reducer was added to the supernatant to reduce iron(III) to iron(II). After a 30-minute incubation at room temperature, iron probe was added to the samples and incubated for another 60 min in the dark at room temperature. Absorbance was determined at O.D. 593nm using EnSpire Multilabel Reader 2300. The intracellular iron content was calculated using the standard curve and normalized to the total number of cells.

### Immunoblotting

Cells were harvested by centrifugation and washed with PBS. Whole-cell lysates were resuspended in PRO-PREP protein extraction solution (iNtRON, Taipei, Taiwan). The extracts were collected and protein concentrations were determined using Bio-Rad BCA reagent (Bio-Rad Hercules, CA). A sample (30 μg) of each lysate was subjected to electrophoresis on sodium dodecyl sulfate-polyacrylamide gels and electroblotted onto PVDF membranes. After blocking, the membranes were incubated overnight with primary antibodies in PBS with 0.05% Tween-20 (TBST) at 4°C. Membranes were then washed four times and incubated with horseradish peroxidase (HRP)-conjugated goat anti-mouse or rabbit IgG (Upstate, Billerica, MA) for 2 h. After washing with TBST, the blots were incubated for 1 min with SuperSignal West Pico ECL reagent (Pierce Biotechnology, Rockford, IL), and chemiluminescence was detected by exposure to Kodak-X-Omat film (Eastman Kodak, Rochester, NY). The β-actin signals were used to verify that equal concentrations of protein were loaded in each lane. Band densities were measured using a gel analysis system (BioSpectrumAC Imaging System Vision Work LS software, UVP, Upland, CA).

### Immunocytochemistry

Cells were cultured on chamber slides (Nalgene Nunc International, Naperville, IL) and fixed with 3.7% formaldehyde in PBS. The cells were blocked with 2% normal horse serum, incubated with antibodies against TFEB, LAMP1, or LAMP2 in PBS containing 0.2% Triton X-100 (PBST), and then incubated with fluorescein isothiocyanate (FITC)-conjugated, goat anti-rabbit IgG or rhodamine-conjugated, goat anti-mouse IgG (Sigma). The cells were washed with PBST, mounted using an anti-fade, DAPI-containing mounting medium (Vector Lab, Burlingame, CA), and analyzed by confocal microscopy (Olympus, FV1000D, Tokyo, Japan).

### Lysosomal pH measurements

Lysosomal pH was measured using the ratiometric lysosomal pH probe, LysoSensor Yellow/Blue dextran (Invitrogen). Cells were stained with 1μg/ml LysoSensor at 37°C for 5 min. Cells were then washed, trypsinized, and resuspended in PBS. The fluorescence signals were monitored using an EnSpire Multilabel Reader 2300 with emission wavelengths at 535 nm and 430 nm, and excitation wavelength at 350 nm. The calibration curve was generated by incubating cells with 10 μM monensin/nigericin in 2-(N-morpholino) ethanesulfonic acid (MES) buffer (5 mM NaCl, 115 mM KCL, 1.3 mM MgSO_4_, 25 mM MES) with the pH adjusted to the range of 4.5–6.5 for 5 min prior to LysoSensor staining. The ratio of the emission at 535 nm to that at 430 nm (535/430 ratio) was calculated for each sample. A lower 535/430 ratio indicates a less acidic lysosomal pH. The pH values were obtained from the calibration curve that was plotted against the pH values in MES buffer.

### Cathepsin D activity assay

Cathepsin D activity was measured using the fluorometric cathepsin D activity assay kits from Biovision (Mountain View, CA) as per the manufacturer instructions. Briefly, 200μl cell lysates containing cathepsin-D was used to cleave the synthetic substrate, GKPILFFRLK(Dnp)-D-R-NH2 labeled with 7-methoxycoumarin-4-acetic acid (MCA) to release a fluorescent product after incubation at 37°C for 2 h. The fluorescence signal was quantified using EnSpire Multilabel Reader 2300 at an excitation wavelength of 328 nm and an emission wavelength of 460 nm for cathepsin D.

### Statistical analyses

Three independent experiments were conducted in all studies and all assay conditions were performed in duplicate or triplicate. The data were analyzed using Student’s *t* test and differences were considered significant at a *p* value < 0.05.

## Results

### Chronic iron overload increases intracellular granularity and iron content in TDM cells

**Fig 1:**
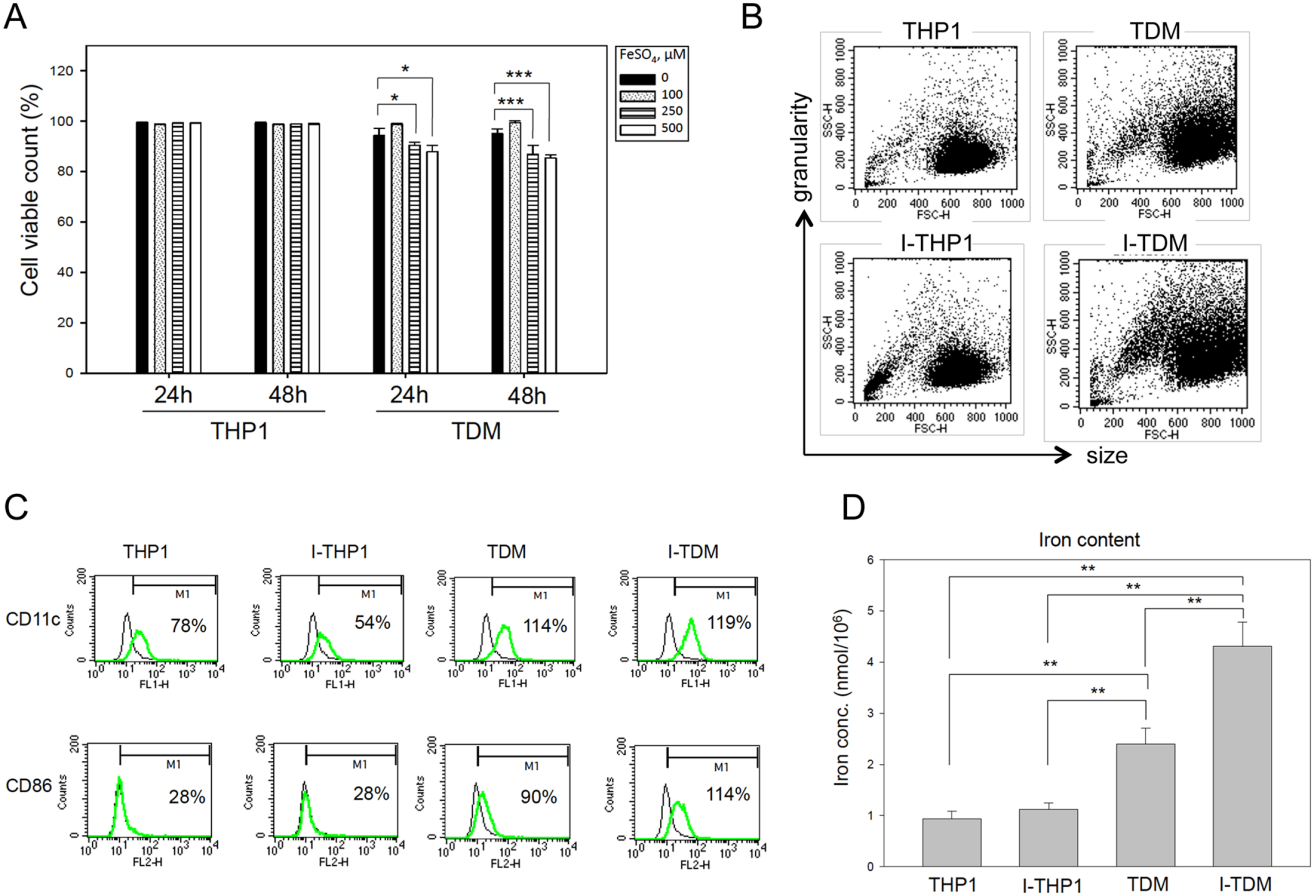
Chronic iron overload increases intracellular granularity and iron content in TDM cells. (A) Cell viability of THP-1 and TDM cells cultured in media with different concentrations of FeSO_4_ for 24 and 48 h was assayed by propidium iodide staining and flow cytometry analysis. (B) After iron exposure, I-TDM cells revealed higher side-scatter intensity than TDM cells, while their forward scatter intensities were comparable. Dot blots are presented for cellular side scatter and forward scatter intensities during flow cytometric analysis of THP-1 and TDM cells with or without chronic iron exposure. (C) A comparative study of CD11c and CD86 expression in THP-1 and TDM cells that cultured under normal iron or chronic iron overload conditions. Flow cytometric analysis of the expression of the indicated surface molecules on THP-1, I-THP-1, TDM, and I-TDM cells was performed. Staining profiles (green lines) are shown for the indicated surface molecules. The thin lines indicate isotype controls. (D) Iron uptake (ferrous and/or ferric ion) of THP-1, TDM, I-THP-1, and I-TDM cells were measured using an Iron Assay Kit. The data represent the average of triplicate experiments and the error bars indicate the standard error of the mean. Significance is denoted as follows: * *p* < 0.05; *** *p* < 0.001.

Macrophage differentiation is associated with presentation of an adhesion phenotype. An increase in cell size and enhanced granularity results from an increase in the number of certain membrane-bound organelles [19]. Consistent with this, differentiated TDM cells showed an adhesion phenotype, with and increased cell size and intracellular granularity compared to THP-1 cells (data not shown). Chronic iron overload of THP-1 cells (I-THP-1) was achieved by subculturing cells persistently in media containing 100, 250, or 500 μM FeSO_4_ that provided 5.6, 14, or 28 μg/ml iron, respectively. Chronic iron overload of TDM (I-TDM) cells was achieved by treating THP-1 cells with PMA for 48 h and culturing differentiated cells persistently in media containing 100, 250, or 500 μM FeSO_4_. The chronic iron overload did not cause a decrease in viability of I-THP-1 cells, but a mild decrease in viability of I-TDM cells at 250 and 500 μM FeSO_4_ (Fig 1A). Serum iron concentration in patients with thalassemia major is between 0.63 and 6.02 μg/mL [20]. Since no toxicity was observed in both tested cell types at iron concentrations close to that observed in clinical iron overload conditions, I-THP-1 and I-TDM cells incubated with 100 μM FeSO_4_ were used in following experiments. After treatment with 100 μM FeSO_4_, chronic iron overload did not change the morphology or size of the I-THP-1 cells, but an increase in intracellular granularity was detected in I-TDM cells only, using flow cytometry (Fig 1B). Activated monocytes/macrophages upregulate surface marker of CD11c and CD86 for cell migration and costimulation deliver to naïve T cells. In the results, chronic iron exposure did not significantly affect CD11c and CD86 expression in THP-1 compared with I-THP-1 cells or TDM compared with I-TDM cells (Fig 1C). Finally, iron storage measured by the Iron Assay Kit revealed that TDM cells contained more iron than THP-1 cells under normal conditions. Additionally, though iron storage was unaffected in I-THP-1 cells, in I-TDM cells increased significantly with chronic iron exposure (Fig 1D). Thus, we conclude that chronic iron exposure increased intracellular granularity and iron content in TDM cells but not in undifferentiated THP-1 cells.

### Chronic iron exposure decreases the bactericidal efficiency of TDM, RAW264.7 cells and human primary monocyte-derived macrophages

**Fig 2:**
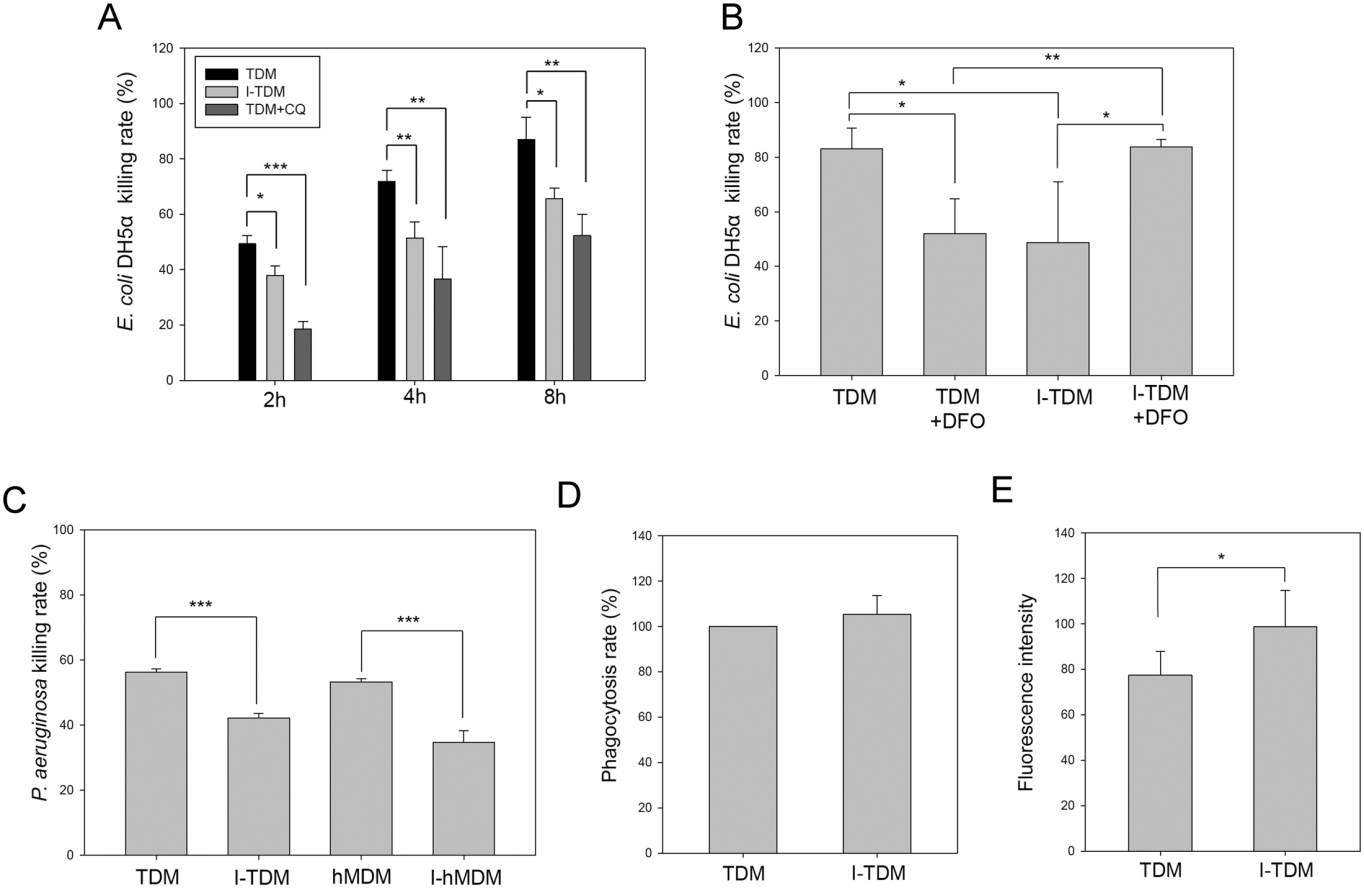
Chronic iron overload impairs the bactericidal activity of macrophages. (A) The bactericidal activity assay was performed by incubating TDM cells, I-TDM cells, and 50μM chloroquine (CQ)-pretreated TDM cells with *E*. *coli* DH5α at 37°C for 0, 2, 4 and 8 h. The bacterial killing rate is presented as the percentage of colony forming units (CFUs) released from cells without incubation (0 h) to the CFUs released from cells sampled at different time points of incubation. (B) The bactericidal activity assay was performed by incubating untreated and DFO-pretreated (100 μM for 12 h) TDM and I-TDM cells with *E*. *coli* DH5α at 37°C for 4 h. (C) The bactericidal activity assay was performed by incubating TDM, I-TDM cells, hMDM and I-hMDM with *Pseudomonas aeruginosa* at 37°C for 4 h. (D) The phagocytic activity of TDM and I-TDM cells was assessed by measuring the phagocytosis of labeled *E*. *coli* BioParticles. (E) TDM and I-TDM cells were harvested and incubated with 10 μM DCFDA for 15 min and then analyzed by flow cytometry. The data represent the average of triplicate experiments and the error bars indicate the standard error of the mean. Significance is denoted as follows: * *p* < 0.05; ** *p* < 0.01; *** *p* < 0.001.

After THP-1 cells differentiated into TDM cells, the bactericidal ability of TDM and I-TDM cells was determined after incubation with non-pathogenic DH5α or JM109 strain of *E*. *coli* for 0, 2, 4 and 8 h. All I-TDM cells showed a significantly decreased bacterial killing rate compared with TDMs (Fig 2A and S1A Fig). The decline in bactericidal ability of I-TDM cells was related to higher intracellular iron content (Fig 1D), which could be restored by treating I-TDM with deferoxamine (DFO), an iron chelator that expels iron from I-TDM cells (Fig 2B). In addition to TDMs, *E*. *coli* DH5α killing ability of the mouse macrophage cell line, RAW264.7 assessed at different time points also showed a decline after culture in medium containing iron (S1B Fig). The pathogenic *Pseudomonas aeruginosa* and primary human monocyte-derived macrophages (hMDM) were further used to do the test and again, the results showed hMDM and TDM cells decrease pathogenic bacterial killing ability after culture in iron-contained medium (Fig 2C).

Defects in phagocytosis of macrophages are associated with increased susceptibility to infection. A phagocytosis assay was used to evaluate whether chronic iron exposure affected the phagocytic ability of TDM cells and the results showed no effect (Fig 2D). Respiratory burst, the rapid release of ROS from macrophages, plays an important role in phagocytes to degrade internalized particles and bacteria. Next, we used the cell permeant reagent 2’,7’–dichlorofluorescin diacetate (DCFDA) to measure ROS within the cell. DCFDA is oxidized by ROS into the highly fluorescent 2’, 7’–dichlorofluorescein (DCF) detected by flow cytometry after diffusion into cells. Although chronic iron exposure decreased the bactericidal ability of TDM cells, I-TDM cells produced more ROS than TDM cells (Fig 2E). Thus, chronic iron exposure decreases the bactericidal efficiency of TDM cells and this defect is not caused by the impairment of phagocytosis or ROS generation.

### Chronic iron exposure causes impairment of lysosomal acidification and lysosomal dysfunction, which results in decreased bactericidal efficiency of TDM cells

**Fig 3:**
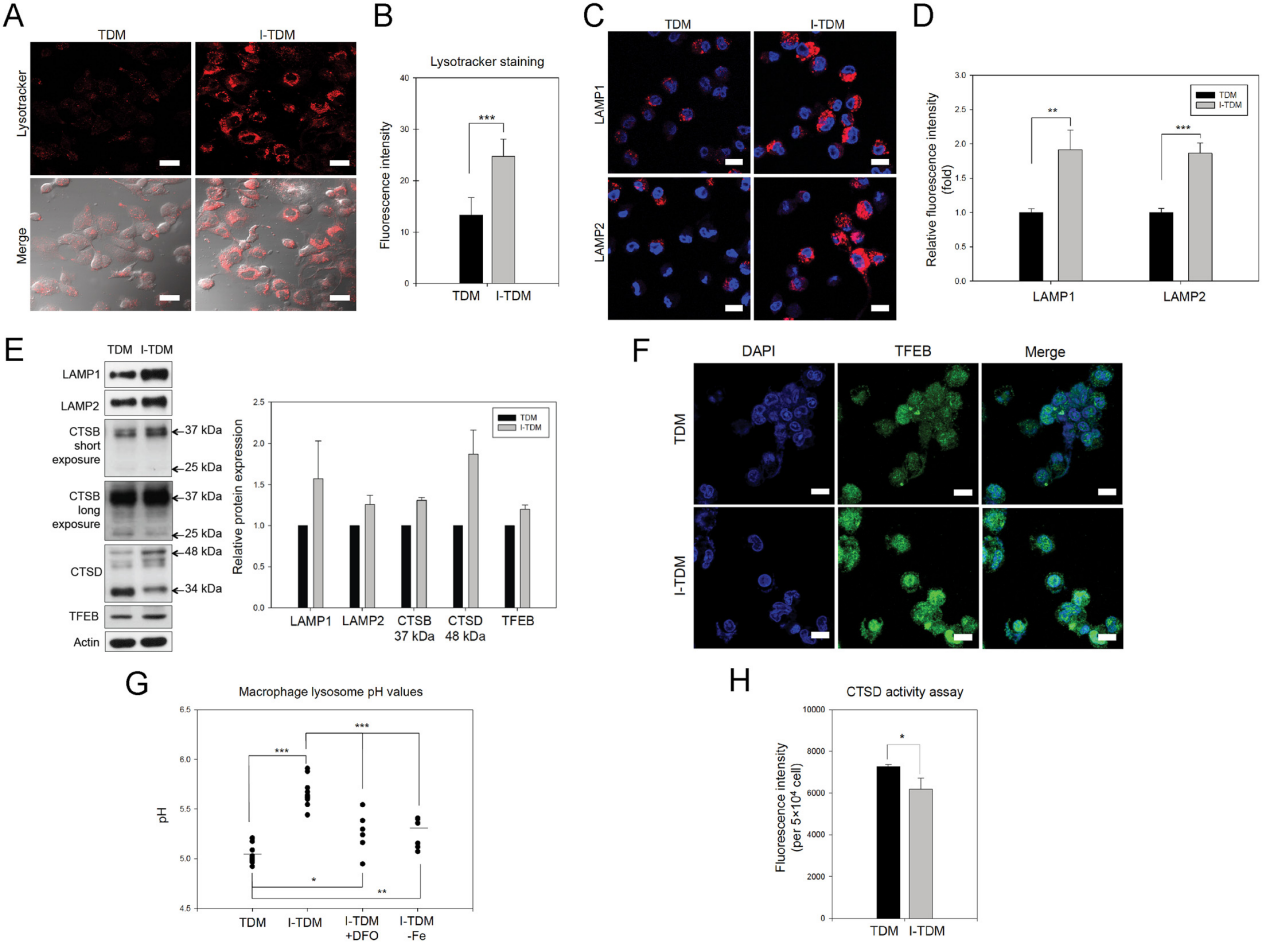
Chronic iron overload increases the total number of lysosomes, reduces lysosome acidification, and results in lysosomal dysfunction in TDM cells. (A) Representative images of TDM cells in the presence or absence of chronic iron overload were obtained by LysoTracker staining and confocal microscopy. (B) The intensity of LysoTracker Red fluorescence was quantified by flow cytometry. The results indicated that the number of lysosomes increased in TDMs following chronic iron exposure. (C) Representative images showed immunofluorescence staining of LAMP1 and LAMP2 in TDM cells with or without chronic iron overload. (D) The intensity of red fluorescence due to immunofluorescence-stained LAMP1 and LAMP2 was quantified by flow cytometry and the results indicated that the number of lysosomes increased in TDMs following chronic iron exposure. (E) LAMP-1, LAMP2, immature cathepsin B, cathepsin D, and TFEB proteins were significantly increased following chronic iron overload of TDM cells. The cell lysates of TDM and I-TDM cells were used for immunoblot analyses with actin as loading control. The results are shown in the right panel. Bars, mean± SEM. (F) The nuclear localization of TFEB proteins in I-TDM cells. TDM and I-TDM cells were fixed with paraformaldehyde, and the fixed cells were incubated with rabbit anti-TFEB antibodies, and with goat anti-rabbit FITC secondary antibody. Scale bars, 20 μm. (G) The lysosomal pH in TDM, I-TDM, I-TDM cells treated with DFO and I-TDMs incubated in iron-free media was quantified using LysoSensor Yellow/Blue dextran and fluorescence ELISA reader. (H) Cathepsin D activity was measured in TDM and I-TDM cells using a fluorometric cathepsin D activity assay. The data represent the average of triplicate experiments and the error bars indicate the standard error of the mean. Significance is denoted as follows: * *p* < 0.05; ** *p* < 0.01; *** *p* < 0.001.

Defects in phagocytosis and ROS generation are not the only possible causes of decreased bactericidal activity in macrophages. Other defects such as lysosomal dysfunction could also have the same effect. After chronic iron exposure, LysoTracker staining (Fig 3A and 3B) and detection of lysosomal membrane proteins, LAMP-1 and LAMP-2 (Fig 3C, 3D and 3E) revealed that I-TDM cells alter lysosomal homeostasis by increasing the number of lysosomes within the cell. Furthermore, I-TDM cells showed an increase in expression of TFEB, a master gene for lysosomal biogenesis [21, 22] and enhanced TFEB translocation into the nucleus (Fig 3E and 3F), which resulted in increased lysosomal biogenesis. A similar phenomenon was found with chronic iron overload of RAW264.7 cells (S2A and S2B Fig). However, lysosomal acidification was significantly impaired measured using lysosomal pH sensors although the increase in number of lysosomes in I-TDMs (Fig 3G). The lysosomal pH increased from 5.0 to 5.7 after chronic iron overload in TDM cells. As the defected bactericidal ability of I-TDM cells was restored by DFO (Fig 2B), DFO treatment and transfer of I-TDM cells to fresh, normal medium partially reversed the inhibition of lysosomal acidification, where lysosomal pH decreased from 5.7 to 5.3 (Fig 3G). Chloroquine, known to elevate lysosomal pH [23], was used to determine if lysosomal alkalization was a main reason for decreased ability of bacterial killing. As shown in Fig 2A and S1 Fig, impaired lysosomal function by chloroquine also led to a decline in bacterial killing rate. Impaired lysosomal function of I-TDM cells was further confirmed by the impaired post-translational processing of cathepsin B and cathepsin D (Fig 3E). With chronic iron overload, TDM cells increased expression of cathepsin B and D intermediate chains (37 and 48 kDa, respectively) but decreased that of mature chains (25 and 34 kDa, respectively). Additionally, cathepsin D activity was also decreased in TDM cells after chronic iron exposure (Fig 3H). Based on these results, we conclude that chronic iron exposure enhances lysosomal biogenesis but impairs lysosomal acidification of TDMs, which leads to decreased bactericidal efficiency.

### Chronic iron exposure blocks autophagolysosome degradation

**Fig 4:**
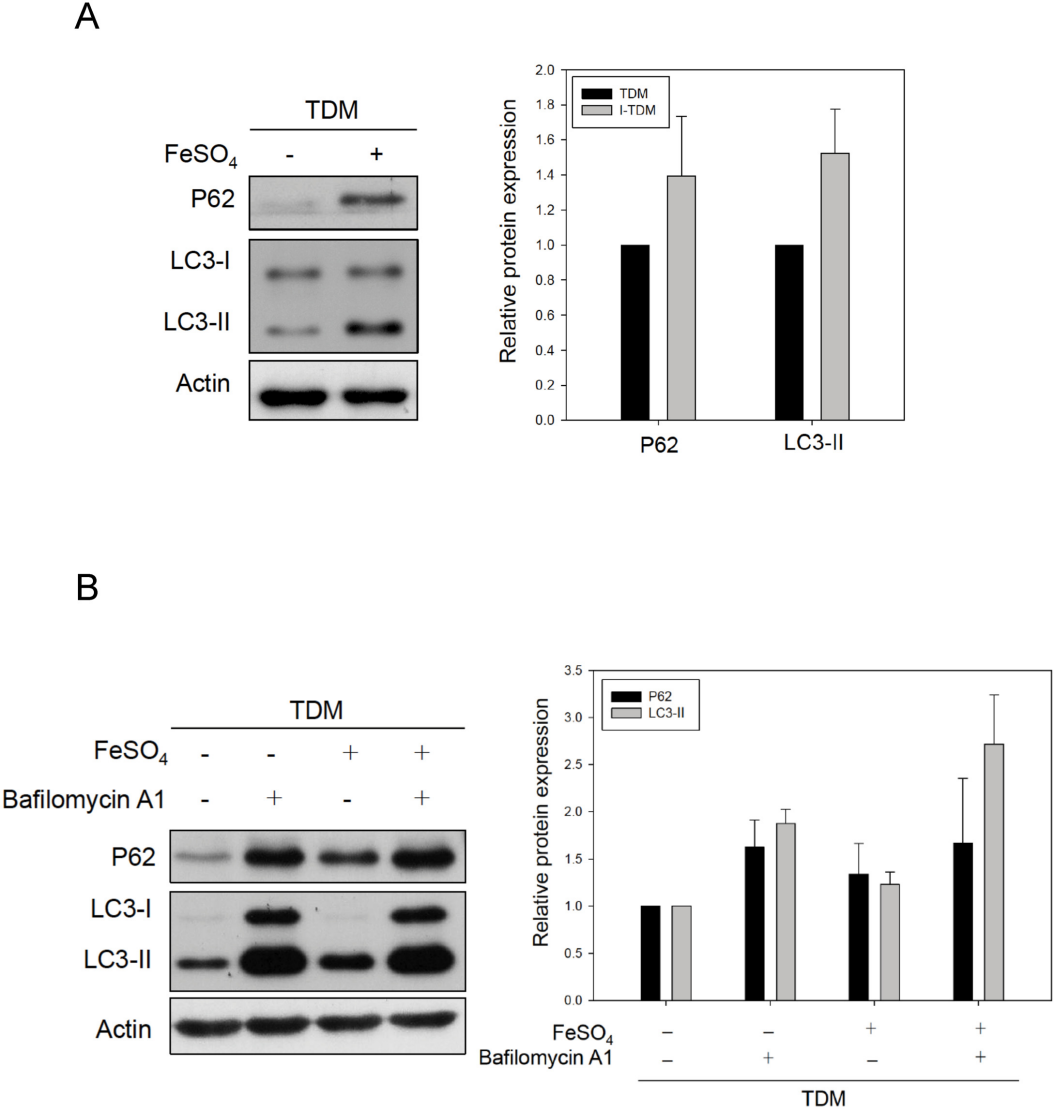
Chronic iron overload disrupts the autophagic flux in TDM cells. (A) The protein levels of LC3-II and p62 in TDM cells with or without chronic iron overload were examined by immunoblotting. (B) In the presence of 10 μM BafA1, chronic iron overload of TDM cells did not increase LC3-II and p62 levels compared with LC3-II and p62 levels in TDM cells treated with BafA1 alone. Actin served as loading control. The quantification results are shown in the right panel. Bars, mean± SEM.

Autophagy is routinely assayed by quantifying autophagosomes according to concentration of LC3-II, a protein that specifically associates with autophagosomes and p62, a protein that is degraded by autophagolysosome via complete autophagic flux [15]. In TDM cells, chronic iron exposure caused an increase in the concentration of both, LC3-II and p62 proteins (Fig 4A). This suggests that chronic iron exposure may inhibit the autophagy flux and block autophagolysosomal degradation resulting in the accumulation of LC3-II and p62. We thus demonstrated the effect of chronic iron exposure in the presence of the lysosomal inhibitor bafilomycin A1 (BafA1) that blocks LC3-II destruction [24]. BafA1 treatment in chronically iron-overloaded TDM cells did not result in a significant increase in LC3-II levels compared with BafA1 alone (Fig 4B), indicating that chronic iron exposure does not induce autophagosome synthesis, but rather blocks LC3-II degradation in the autophagolysosome. Thus, we conclude that chronic iron exposure not only inhibits lysosome function, but also blocks autophagolysosomal degradation.

## Discussion

The results of this study demonstrated that chronic iron overload causes lysosomal alkalization, impairs autophagic flux and lysosomal function of THP-1-derived macrophages, and results in decreased bactericidal efficiency. Macrophages undergo heterogeneous, specific differentiation depending on the local tissue microenvironment and at least two polarized macrophages have been defined [25–27]. M1 macrophages act as effector cells in Th1 cellular immune responses, while M2 macrophages are involved in immunosuppression and tissue repair. Polarized macrophages *in vitro* are capable of complete repolarization in response to fluctuating environments [28]. Different polarized macrophages have differing iron content [29]; however, there are no data describing how iron exposure influences macrophage polarization. From the results, we hypothesized that the influence of chronic iron overload originates during the onset of THP-1 differentiation into macrophages. Whether the polarization of a THP-1-derived macrophage under chronic iron overload deviates to a proinflammatory or an anti-inflammatory characteristic is currently unknown and will require further investigation.

Iron is an essential trace element, required by both microbial invaders as well as their host. Accumulating evidence suggests that the frequency and severity of infections by a variety of microbes are higher in individuals with iron overload (owing to iron supplementation, hereditary hemochromatosis, erythrocyte transfusions, or unknown causes) [30–33]. Multiple mechanisms of innate immunity in the host restrict the supply of iron to microbial invaders (iron sequestration) [17, 33, 34]. In patients suffering from iron overload, excessive ferrite exceeds the capacity of iron sequestration which compromises the patients’ bacteriostatic defenses. However, iron sequestration is not the only defense against invading microbes. Cells of the innate immune system are also part of the frontline defense against microbes. In our results, the non-pathogenic and pathogenic bacterial killing ability of TDM cells and hMDM declined after culture in medium containing iron. There is evidence that iron overload compromises the ability of phagocytes to kill microorganisms [10]. Investigations of innate immunity in patients with transfusion-dependent thalassemia have shown dysfunctional bacterial phagocytosis and reduced generation of superoxide and hydrogen peroxide by macrophages [35]. Decreased phagolysosomal fusion in peripheral blood monocytes has been found in patients with transfusion-dependent thalassemia, which is also related to iron overload [36]. Furthermore, oxidative stress from iron overload also destabilizes the secondary lysosomes of the macrophages, compromising their protective role [35].

In this study, we revealed that lysosomes are one of the main target cellular organelles for chronic iron overload in TDM cells. Pathogen degradation by lysosomal digestion is a powerful mechanism to destroy invading pathogens. In this study, defects in lysosomal acidification and function were found in chronically iron-overloaded TDM cells, as evidenced by lysosomal pH evaluation, impaired cathepsin processing, and decreased cathepsin D activity. Iron chelation resulted in the restoration of lysosomal acidification and bactericidal ability of TDM cells. Interestingly, the increased side scatter of I-TDM cells in the flow cytometry analysis implied that these cells have greater internal complexity, LysoTracker Red staining and LAMP1/LAMP2 fluorescence staining of I-TDM cells confirmed that cells experience enhanced lysosomal biogenesis. Thus, it is possible that TDM cells alter lysosomal homeostasis to compensate for compromised lysosome acidification under chronic iron overload conditions.

Lysosomes are no longer being considered as simply cellular disposal organs for degradation but are involved in a plethora of important processes related to health and disease [37–40]. Children with β-thalassemia major have been reported to harbor subclinical atherosclerosis early in life and are at risk for developing premature atherosclerosis [41]. Some thalassemia major patients may eventually suffer cardiac disease or death because of their symptoms [42]. Our finding of defective lysosomal function in chronically iron-overloaded macrophages provides a new mechanism underlying the pathogenesis of complications in thalassemia major patients.

Based on the results of this study, co-treatment with an iron chelator restores the lysosomal acidification and antimicrobial ability of I-TDM cells. In medical practice, chelation therapy for patients with iron overload that leads to the normalization of iron stores has reportedly reduced the incidence of infections [43]. However, desferrioxamine, the iron chelator used has been implicated in the development of opportunistic infections and been reported to enhance virulence of methicillin resistant *Staphylococcus aureus* in patients with iron overload [44–46]. Some pathogenic bacteria and fungi utilize the iron bound by desferrioxamine to promote their growth. Furthermore, Iron chelators were already found to induce autophagic cell death [47] and DFO-related ocular toxicity [48], DFO increased apoptosis of murine leukemic cell lines also have been reported [49]. DFO decreases bactericidal ability in our results (Fig 2B) may attribute to that DFO is toxic to TDM, not I-TDM cells (S3 Fig). Further studies are needed to understand the mechanism of cell damage by DFO and therefore, it would be useful to develop a novel strategy for infection intervention in patients with iron overload. Lysozyme is an inducible marker of macrophage activation [50]. Recently, lysosomal function and biogenesis were reported to be upregulated by mTOR inhibitors and the induction of lysosomal biogenesis in atherosclerotic macrophages was reported to rescue lipid-induced lysosomal dysfunction and downstream sequelae [51, 52]. Our results demonstrate that I-TDM cells showed an increase in expression and activation of TFEB as well as lysosomal biogenesis, which could be to compensate for impaired lysosome activity. Although lysosome impairment may represent only one aspect of the several potential pathomechanisms of infections in patients with thalassemia major, the results from this study indicate that the enhancement and/or restoration of lysosome function may represent a new treatment option for patients with chronic iron overload.

Autophagy is an evolutionarily conserved process that delivers intracellular organelles within double-membrane vesicles (autophagosomes) to lysosomes for degradation and/or recycling [53–55]. In recent years, autophagy has been revealed as one of the most remarkable components of the immune response and intracellular host cell defense machinery [56–58]. Chronic iron overload increases LC3-II and p62 protein levels in TDM cells. In this study, the LC3-II levels in I-TDM cells pre-treated with bafilomycin A1 were not increased, further demonstrating that a non-lethal dose of iron overload disrupts autophagic flux and autophagolysosomal degradation. Thus, in addition to disrupting intracellular host cell defenses, the impairment of autophagy in macrophages may also affect subsequent adaptive immune response mechanism such as antigen presentation. Furthermore, autophagosome-lysosome fusion is essential for the activation of lysosomes [50]; therefore, impaired autophagic flux in I-TDM cells may also contribute to defects in lysosome function. Hence, investigating the autophagic flux blocking mechanism in chronically iron-overloaded of TDMs may present an alternative therapeutic option to increase lysosomal function in patients with thalassemia major.

The results of our study demonstrate that chronic iron overload reduces the bactericidal activity of TDM cells by disrupting lysosome function. Our findings reveal a novel effect of chronic iron overload on macrophages and highlight the role of lysosomes in antimicrobial defenses. Future strategies to manipulate lysosome function may lead to new therapeutic approaches to treat iron overload.

## Supporting Information

S1 Fig Chronic iron overload impairs bactericidal activity of TDM and RAW 264.7 cells. (A) The bactericidal activity assay was performed by incubating TDM cells, I-TDM cells, and 50 μM chloroquine (CQ) pretreated TDM cells with *E*. *coli* JM109 at 37°C incubation for 0, 2, 4 and 8 h. (B) Bactericidal activity assay was performed by incubating RAW264.7 cells, I-RAW264.7 cells (cultured in medium containing 100 μM FeSO_4_), and 50 μM chloroquine (CQ) pretreated RAW264.7 cells with *E*. *coli* DH5 α at 37°C incubation for 0, 2, 4 and 8 h. The bacterial killing rate is expressed as the percentage of CFUs released from cells without incubation (0 h) to that released from cells sampled at different time points of incubation.
(TIF)

S2 Fig Chronic iron overload increases the expression of TFEB and induces the nuclear translocation of TFEB in RAW264.7 macrophage. (A) TFEB was significantly increased following chronic iron overload of RAW264.7 cells. The cell lysates of RAW264.7 cells cultured in iron-free medium or medium containing 100 μM FeSO_4_ (I-RAW264.7) were used for immunoblot analyses with actin served as loading control. The quantification results are shown in the right panel. Bars, mean± SEM. (B) The nuclear localization of TFEB proteins of I-RAW264.7 cells. RAW264.7 and I-RAW264.7 cells were fixed with paraformaldehyde, stained with rabbit anti-TFEB antibodies and then incubated with goat anti-rabbit FITC secondary antibody. Scale bars, 20 μm.
(TIF)

S3 Fig DFO is toxic to TDM but not I-TDM cells. Viability of 100 μM DFO treated TDM and I-TDM cells for 0, 12, 24 and 48 h was assayed by propidium iodide staining and flow cytometry analysis. The data represent the average of triplicate experiments and the error bars indicate the standard error of the mean. Significance is denoted as follows: * *p* < 0.05; *** *p* < 0.001.
(TIFF)
