# Supplementary figures and images for: Chronic Iron Overload Results in Impaired Bacterial Killing of THP-1 Derived Macrophage through the Inhibition of Lysosomal Acidification
Source: PLoS One. 2016 May 31;11(5):e0156713. doi: 10.1371/journal.pone.0156713 (PMC4886970; doi:10.1371/journal.pone.0156713)

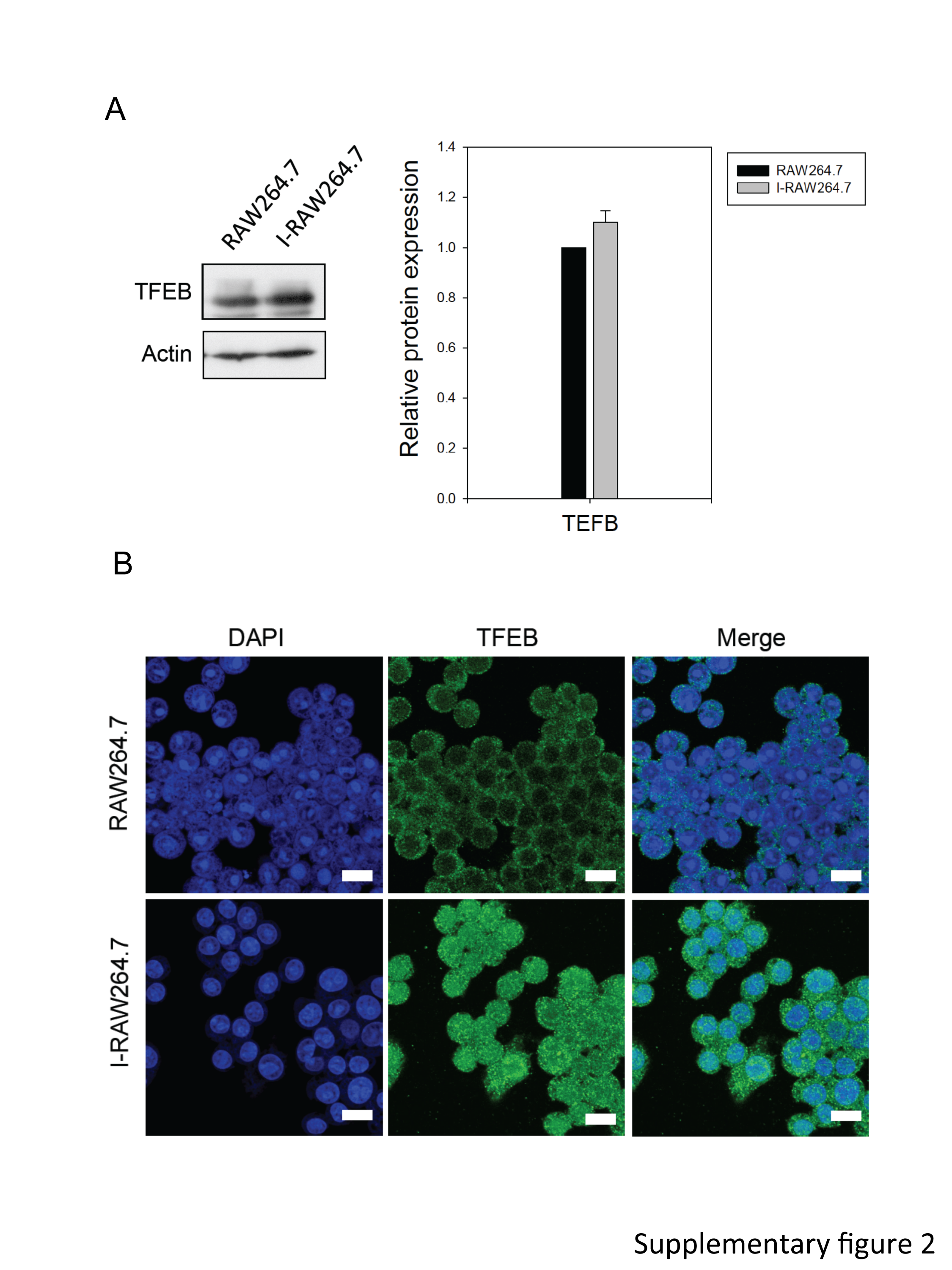

Supplement: S2 Fig — (A) TFEB was significantly increased following chronic iron overload of RAW264.7 cells. The cell lysates of RAW264.7 cells cultured in iron-free medium or medium containing 100 μM FeSO4 (I-RAW264.7) were used for immunoblot analyses with actin served as loading control. The quantification results are shown in the right panel. Bars, mean± SEM. (B) The nuclear localization of TFEB proteins of I-RAW264.7 cells. RAW264.7 and I-RAW264.7 cells were fixed with paraformaldehyde, stained with rabbit anti-TFEB antibodies and then incubated with goat anti-rabbit FITC secondary antibody. Scale bars, 20 μm. (TIF) [file pone.0156713.s002.tif]

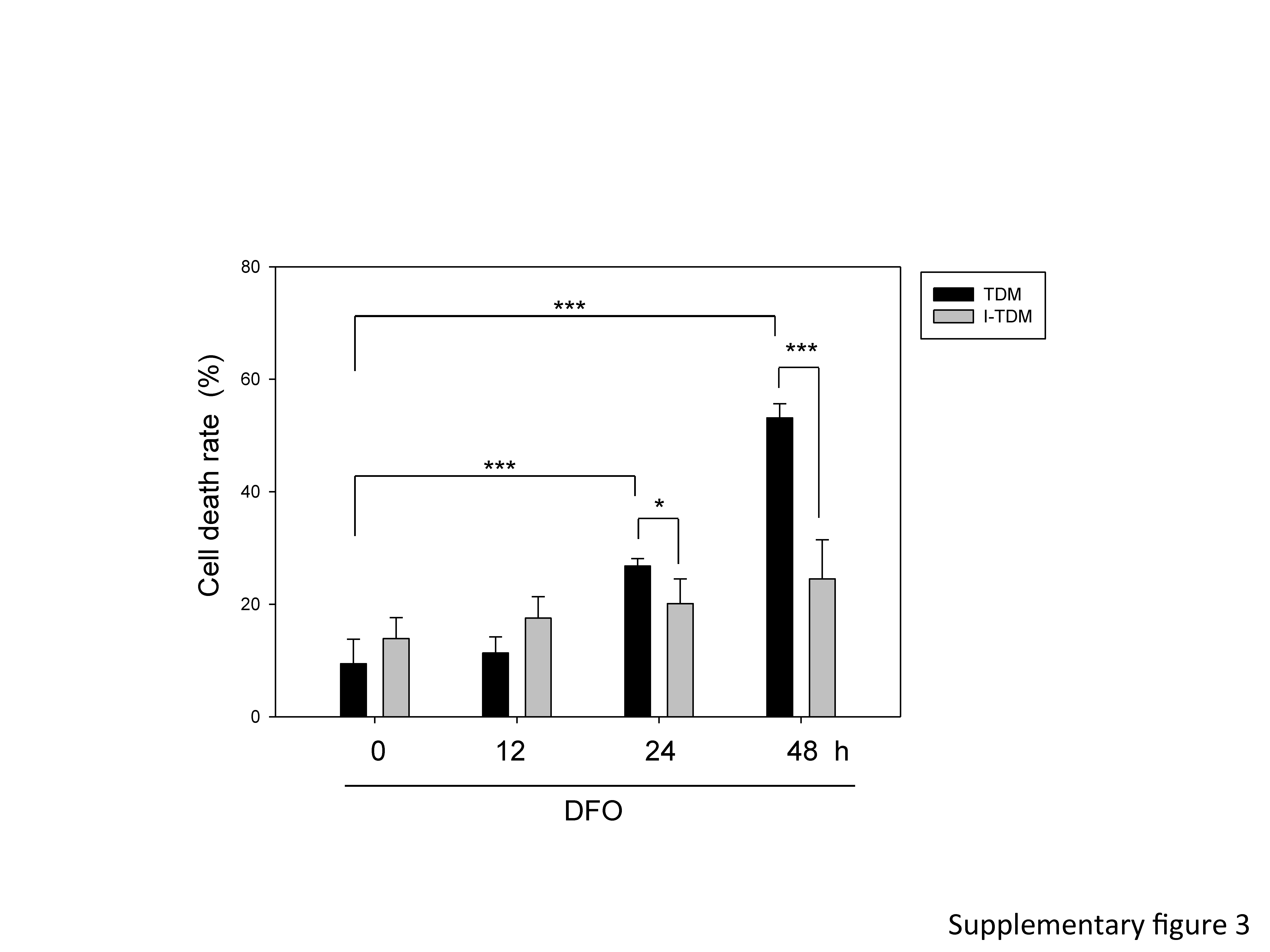

Supplement: S3 Fig — Viability of 100 μM DFO treated TDM and I-TDM cells for 0, 12, 24 and 48 h was assayed by propidium iodide staining and flow cytometry analysis. The data represent the average of triplicate experiments and the error bars indicate the standard error of the mean. Significance is denoted as follows: * p < 0.05; *** p < 0.001. (TIFF) [file pone.0156713.s003.tiff]
